# Discounting Practices in Cost-Effectiveness Analyses in Dentistry: A Systematic Review of Studies Published After 2020

**DOI:** 10.2147/CEOR.S600837

**Published:** 2026-04-18

**Authors:** Ramona Maruster, Michael M Bornstein, Pedram Sendi

**Affiliations:** 1 Department of Oral Health & Medicine, University Center for Dental Medicine Basel, Basel, Switzerland; 2 Division of Clinical Epidemiology, Department of Clinical Research, Basel University Hospital, Basel, Switzerland

**Keywords:** discounting, cost-effectiveness analysis, perspective, time preference, net present value, dentistry

## Abstract

**Background:**

Economic evaluations guide resource allocation decisions in dentistry, where preventive interventions often involve upfront costs and long-term benefits. Discounting can strongly influence results, yet inconsistencies in rate selection and justification persist. Despite growing recognition that discount rate selection substantially influences cost-effectiveness conclusions, no systematic review has focused specifically on discounting practices in dental economic evaluations published under the CHEERS 2022 framework. We therefore reviewed discounting practices in cost-effectiveness analyses (CEAs) of dental interventions published after January 1, 2020.

**Methods:**

We systematically searched eight databases (MEDLINE via PubMed, Web of Science, EconLit, Embase, PROSPERO, Central, Scopus, CEA Registry) for full economic evaluations of dental interventions published after January 1, 2020, with time horizons >1 year. Inclusion was limited to Latin alphabet publications and empirical studies. Backward/forward citation tracking supplemented the search. Reporting quality was appraised using CHEERS 2022. Extracted data included discount rates, justifications, perspectives, model types, and intervention categories.

**Results:**

From 2581 records, 83 studies were included. Discount rates ranged from 1.5% to 9%; 3% was most common (41%). Thirteen percent omitted discounting, and 27% provided no justification; when justified, 60% cited government guidelines. Perspectives were predominantly healthcare sector (44%), with societal only 8%. Preventive interventions accounted for 47%. Average CHEERS 2022 compliance was 80.4%.

**Conclusion:**

Discounting practices in recent dental CEAs remain inconsistent, potentially biasing against preventive strategies. Greater adherence to reporting standards and context-specific guideline updates are needed.

## Introduction

Oral health has recently been brought to the forefront of global health discussions, highlighted by a World Health Organization report describing the global burden of oral diseases and calling for improved funding, more efficient resource allocation, and policy reforms.^1^ In many countries, oral health care accounts for 5–10% of total health expenditure, underscoring policymakers’ need for high-quality evidence on the costs and outcomes of dental interventions.^2^ This need is reflected in the increasing number of economic evaluations conducted in dentistry.^2^,^3^ Although their methodological quality has generally improved, previous reviews consistently report shortcomings related to discounting practices, particularly regarding the rationale for the discount rate selected.^4^,^5^

In medical interventions, both costs and benefits frequently occur in the future, making adjustment for differential timing essential.^6^ Discounting reflects the time value of resources, future costs and outcomes are typically valued less than present ones, and can substantially influence economic evaluation results and, consequently, resource allocation decisions.^6^,^7^ A 3% discount rate has long been recommended for comparability across studies; however, recent research suggests that uniform application of this rate may introduce systematic bias.^8^ In particular, applying a 3% rate globally may overvalue future costs and benefits in low- and middle-income countries by failing to account for higher economic growth rates, where discount rates of 4–6% would be more appropriate.^8^ It may also undervalue interventions with early costs but delayed benefits, such as preventive oral health programs.^9^

The choice of discount rate has been the subject of extensive debate, with increasing recognition that it depends on the decision context considered most relevant.^6^,^10^ Under a fixed health care budget, the appropriate discount rate should reflect the opportunity cost of capital and the expected growth in the cost-effectiveness threshold.^11^ In an unconstrained societal perspective, the rate is derived from the social rate of time preference minus expected growth in the consumption value of health, potentially justifying different rates for costs and health effects.^9^,^12^ A related controversy concerns differential discounting. Proponents argue that if the consumption value of health is expected to rise over time, health outcomes should be discounted at a lower rate,^9^ with empirical support in some high-income contexts.^13^ Critics cite insufficient evidence, the “consistency argument” that a life-year has constant value,^14^ and the Keeler-Cretin paradox.^15^

Discounting guidance remains heterogeneous across jurisdictions. Many national guidelines prescribe equal discounting without explicit justification, while others rely on comparability, opportunity cost, or theoretical arguments.^16^ In recent years, discussions about revising recommended discount rates have intensified, with analyses suggesting that declining real interest rates and lower expected consumption growth justify reducing rates to the 1.5–2% range in high-income settings.^17^ This is particularly relevant for dentistry, where many interventions such as sealants, fluoride varnishes, and caries prevention programs incur upfront costs but yield benefits over decades, similar to vaccinations or screenings.^13^,^17^,^18^ These long-term profiles make discounting decisions highly influential on cost-effectiveness projections, potentially biasing against preventive strategies if rates are too high or uniform.^8^,^10^ Prior dentistry-specific reviews highlight persistent gaps in justifying rates,^4^,^5^ underscoring the need for focused assessment to improve methodological consistency in this field.

With this systematic review, we aim to contribute to the methodological literature on economic evaluations in dentistry by providing a focused assessment of discounting practices in the recent dental literature. Specifically, we examine the discount rates applied, the extent to which their use is justified in relation to the stated study perspective, and how current practices align with contemporary methodological guidance. By doing so, this study seeks to support improvements in the conduct and reporting of economic evaluations in dentistry.

This review differs from existing related systematic reviews in three important ways. First, prior reviews by Tonmukayakul et al (2015),^5^ Nguyen et al (2023),^19^ Hettiarachchi et al (2018),^20^ and Mariño et al (2020)^21^ assessed the general methodological quality of dental economic evaluations but did not focus specifically on discounting practices. Second, by restricting inclusion to post-2020 publications, we were able to assess the degree to which the revised CHEERS 2022 reporting checklist, which strengthened requirements for discounting justification, has been adopted in practice. Third, we explicitly examine the alignment between the stated study perspective and the rationale provided for the discount rate applied.

## Methods

The databases MEDLINE via PubMed, Web of Science, EconLit, Embase, PROSPERO, Central, Scopus and the CEA Registry were searched on November 5, 2024, using a combination of Medical Subject Headings and general search terms. Details of the search strategy and combinations of search terms are provided in the Appendix (Tables S3–S10). We limited the search to empirical cost-effectiveness analyses in dental medicine published after January 1, 2020, to be broadly in line with the CHEERS 2022 checklist and to capture more recent cost-effectiveness methodologies applied in dental research. Further inclusion criteria were a time horizon of more than one year, justifying the discounting of costs and/or effects, and publication in Latin alphabet. In addition, backward and forward citation tracking was performed, followed by web, grey literature, and hand searching. This systematic review was not registered on PROSPERO because it focuses on a methodological/econometric topic in cost-effectiveness analysis without a direct effect on health in humans, which is required for inclusion in PROSPERO.

After removal of duplicates, titles and abstracts were screened independently by two reviewers (R.M. and P.S), with any disagreements resolved by discussion. Eligibility criteria were publication in Latin alphabet and empirical studies evaluating the cost-effectiveness of a dental intervention. When eligibility could not be determined based on title and abstract, the full-text article was screened to assess inclusion. For each included article, we extracted the following study characteristics: author name, year and country of publication, research topic, study type, time horizon, perspective, currency, clinical outcome, whether a discount rate was applied to costs and/or effects, the value of the discount rate, and how its use was justified and referenced. In addition, the CHEERS 2022 checklist^22^ was applied, and the results are presented as Supplementary Table 2. All included papers were reviewed by two independent reviewers to reduce extraction errors.

## Results

**Figure 1:**
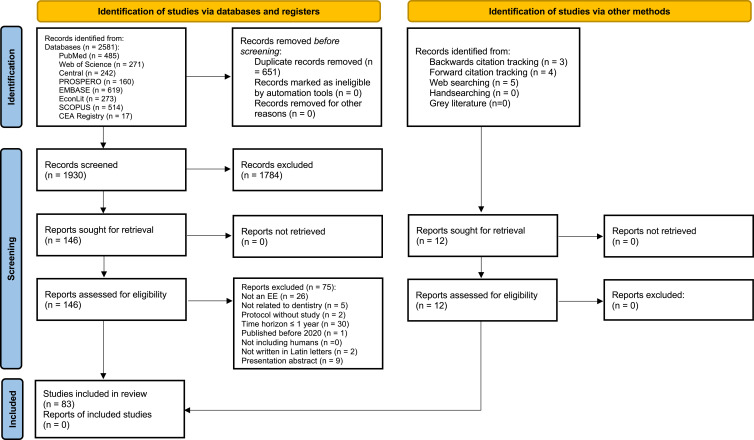
PRISMA 2020 flow diagram.

In total, 2581 articles were identified across eight databases as follows: PubMed (n = 485), Web of Science (n = 271), Central database (n = 242), PROSPERO (n = 160), Embase (n = 619), EconLit (n = 273), Scopus (n = 514), and the CEA Registry (n = 17). After removing 651 duplicates, 1784 studies were excluded during title and abstract screening. Of the remaining 146 articles, 83 were included in this review (Figure 1).^23–106^ Table 1 summarizes the characteristics of the included studies regarding discounting practices and study perspectives. Among the 83 economic evaluations, 11 (13%) did not report any discount rate, and 14 (16%) applied discounting only to costs. Reported discount rates ranged from 1.5% (one study) to 9% (one study), with 3% being the most frequently used rate (34 studies), followed by 3.5% and 5% (14 studies each) (Table 1). Furthermore, 23 studies (27%) did not provide any reference for their decision regarding the application of discount rates (Table 1). Among the studies that did provide a reference, 50 (60%) cited specific government guidelines for reporting economic evaluations (Table 1). The remaining 10 studies (12%) relied on expert advice, previously used discount rates, or bank interest rates (Table 1).

**Table 1:** Study Characteristics of the 83 Included Studies in This Systematic Review

First Author	Year	Perspective	Time Horizon	Discount Rate for Costs in %	Discount Rate for Effects in %	Reference for Discounting Choice	Justification for Discounting Choice
Ahn et al^105^	2022	National health insurance	5 years	0	0	Not reported	Unknown
Almadani et al^41^	2020	Federal health insurance	15 years	0	0	Not reported	Unknown
Anopa et al^78^	2022	Public sector	2 years	1.5	1.5	Government guidelines: NICE 2012	Maximising health gains from a fixed NHS budget
Basu et al^44^	2020	Employer & health care	10 years and lifetime	3	3	Not reported	Unknown
Belotti et al^75^	2024	Societal	20 years	3.5	3.5	Not reported	Unknown
Boachie et al^51^	2023	Public sector health care payer	10 years	5	5	Government guidelines: National department of health	Adjustment for time preference
Brocklehurst et al^71^	2021	Health care system and patient	15 months	3.5	3.5	Government guidelines: NICE 2013	UK Treasury recommendations
Bruhnke et al^83^	2023	Societal & private payer	2.5–5.5 years	3	0	Government guidelines: IQWIG 2009	International long-term equity market costs
Choi et al^52^	2023	Health care	10 years	3	3	Not reported	Unknown
Choi et al^34^	2020	Health care	Lifetime	3	3	Not reported	Unknown
Clarkson et al^80^	2020	Health care system and patient	4 years	0	0	Not reported	Unknown
Clarkson et al^102^	2020	Health care system and patient	4 years	3.5	3.5	Government guidelines: NICE 2013	UK Treasury recommendations
Cronin et al^24^	2021	Health payer	Lifetime	4	0	Government guidelines: HIQA	Society’s time preference
da Costa Rosa et al^56^	2024	Public health care system	10 years	5	5	Government guidelines: REBRATS 2014	For methodological and social reasons
da Silva et al^38^	2020	Dental CEO	10 years	5	5	Government guidelines: REBRATS 2014	For methodological and social reasons
Davidson et al^89^	2022	Health care	7 years	3	0	Government guidelines: LFNAR 2003	Unknown
Davoodi-Lahijan et al^76^	2021	Provider	4 years	5	5	Not reported	Unknown
Durham et al^69^	2021	Health care system	Lifetime	3.5	3.5	Government guidelines: NICE 2020	UK Treasury recommendations
de Medeiros Neto et al^39^	2024	Private practice	15 years	5	0	Previously used rates	Unknown
Effenberger et al^66^	2022	Payer	2 years	4.5	0	Water research commission report	International benchmarks and marginal return on capital
Egil et al^63^	2023	Payer & health care system	10 years	3	3	Panel on Cost-Effectiveness in Health and Medicine	Economic growth, consumption rate of interest, comparability across studies
Emara et al^91^	2020	Mixed public/private payer	Lifetime	3	3	Government guidelines: IQWIG 2009	International long-term equity market costs
Frankenberger et al^84^	2022	Patient	50 years	0	0	Not reported	Unknown
Garbim et al^23^	2024	Payer	2 years	0	0	Not reported	Unknown
Goodwin et al^79^	2022	Health care sector and local authority	5 and 6 years	3.5	3.5	Government guidelines: NICE 2013	UK Treasury recommendations
Gupta et al^94^	2021	Insurance	63 years	0	0	Not reported	Unknown
Halasa-Rappel et al^98^	2021	Payer & societal	5 years	3	3	Not reported	Unknown
Han et al^53^	2024	Patient, limited societal and health care system	Lifetime	4.5	0	Korean Guidelines for Pharmacoeconomic Evaluations 2021	Rate used in preliminary feasibility study on public investments
Homer et al^49^	2020	Health care provider	2–3 years	3.5	3.5	Government guidelines: NICE 2013	UK Treasury recommendations
Hounsome et al^100^	2020	Health care system	80 years	3.5	3.5	Government guidelines: NICE 2013	UK Treasury recommendations
Innes et al^32^	2024	Health care system	2.5 years	Unknown	Unknown	Not reported	Unknown
Janusz et al^26^	2024	Health care sector and limited societal	17 years	3	3	US Panel on Cost-effectivenss in Health and Medicine 2016	Real consumption rate of interest and economic growth and to ensure backward comparability
Jardim et al^59^	2023	Unknown	5 years	9	0	Federal bonds’ interest rate	Federal bonds’ interest rate
Jevdjevic et al^81^	2021	Societal	10 years	3	3	Government guidelines: IQWIG 2019	Not mentioned
Kanzow et al^90^	2021	Mixed public-private payer	Variable	3	0	Government guidelines: IQWIG 2009	International long-term equity market costs
Kularatna et al^70^	2020	Health care system	10 years	5	5	Medical Services Advisory Committee Technical Guidelines 2016	Not mentioned
Lamu et al^86^	2022	Societal	2 years	4	4	Government guidelines: NOU 2012	Risk free interest rate and time horizon (4% 0–40 years, 3% 40–75 years, 2% afterwards)
Losenická et al^60^	2021	Patient	30 years	3	3	Government guidelines: CFES 2020	Inflation expectation and society preference
Lukssamijarulkul et al^57^	2022	Health care provider	5 years	0	0	Not reported	Unknown
Maguire et al^33^	2020	Health service provider	2–3 years	3.5	3.5	Government guidelines: NICE 2013	UK Treasury recommendations
Marshman et al^31^	2024	Health care system	2.5 years	3.5	3.5	Government guidelines: NICE 2022	UK Treasury recommendations and consistency accros the public sector investment decisions
Martins et al^64^	2023	Private payer	Lifetime	4	1.5	Government guidelines: Dutch National Health Care Institute 2016	To account for the growing value of health benefits in the future
Matthys.et al^65^	2020	Payer	5 years	4	1.5	Government guidelines: CVZ 2004 and 2006	Expected increase in the value of health benefits
Naved et al^101^	2025	Mixed insurance and patient	5 years and lifetime	3	3	Not reported	Unknown
Naved et al^37^	2024	Private payer	Lifetime	3	3	Expert advice: Haacker et al 2022: US Panel on Cost-effectiveness	Real consumption rate of interest and economic growth and to ensure backward comparability
Naved et al^85^	2024	Private payer	Lifetime	3	3	Expert advice: Haacker et al 2022: US Panel on Cost-effectiveness	Real consumption rate of interest and economic growth and to ensure backward comparability
Nguyen et al^92^	2023	Societal & health care	10 years and lifetime	3	3	Previously used rates	Previously used rates
Nguyen et al^27^	2020	Health care system	70 years	5	5	Government guidelines: PBAC 2016	Present value reflection of future costs
Nguyen et al^73^	2024	Health care system	18 months	0	0	Not reported	Short time horizon
Norrie et al^35^	2020	Regional health authority	5 years	3	3	Not reported	Closest value to the Canadian inflation rate
Okubo et al^68^	2023	Payer	2 years	3	3	Not reported	Unknown
Olegário et al^87^	2020	Payer	2 years	0	0	Not reported	The values were updated
Olegário et al^29^	2022	Public health care system	2 years	0	0	Not reported	Unknown
Pires et al^99^	2021	Public health care system	9 years	6.5	6.5	Brazilian Central Bank 2019	Short-term interest rate
Queiroz et al^72^	2020	Social services	15 and 25 years	5	5	Government guidelines: REBRATS 2014	For methodological and social reasons
Riley ei al.^97^	2020	Health care system	Lifetime	3.5	3.5	Government guidelines: NICE 2013	UK Treasury recommendations
Rodriguez et al^67^	2022	Public provider	4 years	3	3	Government guidelines: MINSAL 2013	Rate of return on long-term government investments
Rojas-Gómez et al^95^	2022	Third-party payer	5 years	5	5	Government guidelines: IETS, 2014	Recommended by the Finance and Economy Ministries
Rossi et al^47^	2022	Payer	Lifetime	3	3	Government guidelines: IQWIG 2009	International long-term equity market costs
Sanghvi et al^103^	2023	Unknown	Lifetime/62 years	3.5	3.5	Government guidelines: NICE 2019	UK Treasury recommendations
Schwendicke et al^88^	2021	Mixed public/private payer	Lifetime	3	0	Government guidelines: IQWIG 2009	International long-term equity market costs
Schwendicke et al^58^	2023	Mixed public/private payer	Lifetime	3	3	Government guidelines: IQWIG 2009	International long-term equity market costs
Schwendicke et al^30^	2022	Mixed public/private payer	Lifetime	3	0	Government guidelines: IQWIG 2017	Rate of inflation
Schwendicke et al^46^	2022	Mixed public/private payer	Lifetime	3	0	Government guidelines: IQWIG 2009	International long-term equity market costs
Schwendicke et al^48^	2021	Mixed public/private payer	Lifetime	3	3	Government guidelines: IQWIG 2009	International long-term equity market costs
Schwendicke et al^54^	2021	Payer	3 years	3 and 5	0	Expert advice and government guidelines: Croatian HTA 2002	Reflecting the trend in the base rate and discount rate in the last years
Schwendicke et al^82^	2021	Payer	3 years	3	0	Government guidelines: IQWIG 2009	International long-term equity market costs
Sharda et al^93^	2025	Societal	65 years	3	3	National guidelines: HTAIn 2023	For study comparability, most recommended and applied value globally and in India
Souto et al^104^	2021	Public health care system	30 years	5	5	Government guidelines: REBRATS 2014	For methodological and social reasons
Stanley et al^50^	2020	Health care sector	Lifetime	3	3	US Panel on Cost-effectivenss in Health and Medicine 2016	Real consumption rate of interest and economic growth and to ensure backward comparability
Tang et al^43^	2024	Social	3 years	3	3	Not reported	Adjustment for inflation
Tannous et al^77^	2021	Health healthcare provider	4 years	0	5	Government guidelines: PBAC 2016	Present value of future costs
Taylor^106^	2022	Health care system	Lifetime	3.5	3.5	Government guidelines: NICE 2022	UK Treasury recommendations and consistency accros the public sector investment decisions
Tekpınar et al^36^	2024	Societal	20 years	5	5	Expert advice: M. Haacker, Department of Global Health and Population, Harvard	To account for the economic growth of lower-midle-income countries
Victory et al^40^	2022	Health care system	2 years	3.5	3.5	Government guidelines: NICE 2013	UK Treasury recommendations
Werbrouck et al^96^	2022	Payer	10 years	3	1.5	Government guidelines: KCE 2008	Return of risk free bonds and the change in the value of health over time
Whittaker et al^74^	2024	Health care system	5–6 years	3.5	3.5	Government guidelines: NICE 2013	UK Treasury recommendations
Zang et al^42^	2023	Societal	5 years	5	0	Not reported	Unknown
Zaror et al^45^	2020	Public Payer	2 years	3	3	Government guidelines: Health Ministry of Chile 2013	For consistency and comparability with other studies and to promote prevention programs
Zhao et al^62^	2023	Health sector	20 years	2.1	3.4	Not reported	General and health inflation
Zhou et al^55^	2023	Societal & payer	5 years	3	3	WHO Guide 2003	For comparability across studies
Zhu et al^25^	2024	Health care system	20 years	5	5	Government guidelines: China Guideline for Pharmacoeconomic Evaluations 2020	Reflects socioeconomic growth, fluctuations in prices and preferences of consumers
Zhurakivska et al^61^	2023	Patient	3 years	3	3	Government guidelines: NICE 2013	UK Treasury recommendations

Regarding the perspectives adopted, 2 of the 83 studies did not specify a perspective, 21 (25%) used a mixed perspective, 7 (8%) used a societal perspective, 16 (19%) adopted a patient/payer perspective, and 37 (44%) reported a health-care perspective (Table 1). A detailed list of study characteristics, including year and country of publication, time horizon, and currency, is provided in Table S1 in the Appendix. With respect to the cost-effectiveness methodology used, 66% of the included articles used a Markov model, and almost half (47%) focused on various preventive dental interventions. The results of the CHEERS 2022 checklist are reported in Table S2 in the Appendix. An average reporting compliance of 80.4% was achieved according to the CHEERS 2022 criteria.

## Discussion

Of the 83 articles included in this review, 13% did not report using a discount rate, and 27% did not provide any reference to justify their choice. The most frequently used value for discounting was 3%, applied in 41% of the studies, consistent with earlier findings.^8^,^107^ These studies were conducted predominantly in high-income countries and showed no consistent preference for a particular analytic perspective. This lack of consistency in economic evaluations regarding perspective choice has also been highlighted previously.^108^,^109^ Moreover, 24% of the studies using a 3% rate did not provide any reference for their choice, while the rest almost exclusively relied on government guidelines. In contrast, the 3.5% rate was used almost entirely in UK-based studies, all of which applied a health-care perspective. Studies adopting a 5% discount rate were largely conducted in middle-income countries and predominantly used a health care perspective. Higher discount rates, such as 6.5% and 9%, were observed only in Brazil, again aligned with a health care perspective. Finally, differential discounting was used in the Netherlands and Belgium, where outcomes were discounted at a lower rate than costs, consistently within a payer perspective.

The findings of this review are consistent with the broader landscape of oral health economic evaluation research, which shows a steady increase in the number of studies alongside ongoing methodological heterogeneity.^4^,^5^ Previous reviews have similarly documented a strong focus on dental caries, frequent omission of discounting in multi-year analyses, and variability in the application of discount rates.^4^,^8^,^19–21^ The predominance of the 3% rate aligns with traditional recommendations for comparability across studies.^8^ However, our post-2020 sample reveals persistent discussions around uniform rates, particularly in low- and middle-income countries where higher rates have been proposed to better reflect economic growth.^8^ Unlike earlier reviews that emphasized methodological shortcomings, our analysis indicates improved overall reporting quality, likely influenced by updated standards.^110^

The choice of discount rate is context-dependent.^12^ Under a fixed health care budget, it should reflect the opportunity cost of capital and expected growth in the cost-effectiveness threshold.^11^ In an unconstrained societal perspective, the discount rate for costs equals the social rate of time preference minus expected growth in the consumption value of health, potentially justifying different rates for costs and health effects.^6^,^12^ Differential discounting remains controversial: proponents argue for a lower rate for health outcomes if the consumption value of health rises over time (with some empirical support in high-income settings), while critics cite insufficient evidence, the consistency argument (constant value of a life-year), and paradoxes such as the Keeler-Cretin effect.^13^,^15^ Guidance remains heterogeneous, with many guidelines prescribing equal discounting without explicit justification.^28^,^107^,^108^ Recent discussions suggest reducing rates to 1.5–2% in high-income settings due to declining real interest rates and consumption growth.^17^ This is particularly relevant for dentistry, where upfront costs and decades-long benefits make discounting highly influential, often biasing against preventive strategies under uniform or high rates.^4^,^9^ Prior dentistry-specific reviews highlight persistent gaps in justification, underscoring the need for focused assessment.^5^,^20^

This review has several strengths. Its recency—limited to studies published after January 1, 2020, allows it to reflect contemporary practices in the context of the latest CHEERS guidance and PRISMA recommendations, providing timely insights amid rising global attention to oral health burdens.^1^,^22^,^111^ The inclusion of 83 studies exceeds the sample sizes of previous dentistry-specific reviews, ensuring greater comprehensiveness through systematic multi-database searching and rigorous assessment of reporting quality using the CHEERS 2022 statement.^4^,^5^ This approach identified high average reporting compliance (80.4%) and revealed policy-relevant patterns, such as potential bias against preventive interventions when uniform or high rates are applied without justification.^9^,^19^ This review builds on earlier work by emphasizing the role of discounting in long-term modelling, documenting guideline heterogeneity, and advocating consideration of context-specific rates. These elements enhance its relevance for improving resource allocation, particularly in preventive programs in low- and middle-income settings.^8^

The policy implications of inconsistent discounting practices are particularly consequential for preventive dental interventions. Programs such as fissure sealants, fluoride varnishes, and community water fluoridation incur costs almost entirely at the beginning of the treatment, while their benefits accrue over decades, similar to vaccination or screening programs. Applying a uniform or elevated discount rate in settings where a lower rate would be appropriate based on the social rate of time preference systematically disadvantages these interventions in cost-effectiveness rankings, potentially biasing resource allocation decisions against prevention. We therefore recommend that guideline-issuing bodies in dentistry explicitly require sensitivity analyses on the discount rate, particularly for preventive interventions with time horizons exceeding 10 years, and that researchers clearly align their rate selection with the stated analytic perspective and the economic context of the study country.

Limitations, while present, are addressed through the study design. The restriction to post-2020 publications may exclude historical patterns, although this was deliberate to reflect current methodological standards. Language restrictions (Latin alphabet only) may have under-represented studies from certain regions, particularly low- and middle-income countries. Although the primary aim of this review was to document and analyze discounting practices in relation to perspective selection, other methodological aspects such as the measurement and valuation of outcomes or the application of sensitivity analysis, were not examined in depth, as these have been addressed in previous publications.^112^ In addition, as this review was intentionally focused on dental medicine, the findings are specific to this field and may not directly generalize to other areas of health care.

## Conclusions

Based on the findings of this study, we recommend that researchers in the dental field adhere more consistently to established reporting standards and, when selecting discount rates, carefully consider and explicitly document the rationale underlying the guidelines they choose to follow.^22^,^107^ Discounting decisions should be aligned with other core study parameters, including analytic perspective, time horizon, and outcome measure.^6^,^12^ Further work is needed to identify the main drivers of inconsistency in discounting practices and to support the development of more up-to-date, comprehensive guidelines that incorporate relevant methodological and contextual factors, thereby enhancing the comparability and policy relevance of economic evaluations in oral health. In particular, the persistent use of uniform discount rates across diverse country contexts, and the frequent absence of theoretical justification, risks introducing systematic bias against preventive dental interventions whose cost-effectiveness is highly sensitive to long-term discounting assumptions. Dental HTA bodies should therefore consider revisiting their discounting guidance in light of recent methodological developments and the updated CHEERS 2022 reporting standards, with explicit attention to the alignment between rate selection, analytic perspective, and country-specific economic parameters.

## Data Availability

No new data were generated or analyzed in this study; therefore, data sharing is not applicable.
